# Supplementary material for: Causal Relationship between Aging and Anorexia Nervosa: A White-Matter-Microstructure-Mediated Mendelian Randomization Analysis
Source: Biomedicines. 2024 Aug 16;12(8):1874. doi: 10.3390/biomedicines12081874 (PMC11351342; doi:10.3390/biomedicines12081874)
Supplement: Supplementary file 1 [file biomedicines-12-01874-s001.zip › Supplementary Figure/Figure S8/Leaveoneout plot of MR analysis for TL to ubm-b-1966.pdf]

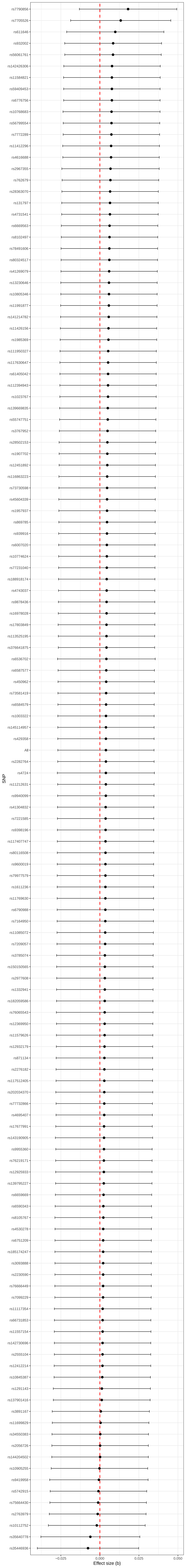
rs7790856
rs7705526
rs611646
rs932002
rs56061761
rs142426306
rs11584821
rs59409453
rs6776756
rs10768683
rs56799554
rs7772289
rs11412296
rs4616688
rs2967355
rs762679
rs28363070
rs131797
rs4731541
rs6669563
rs8102497
rs78491606
rs80324517
rs41269079
rs13230646
rs10805346
rs11991877
rs141214782
rs11426156
rs1985369
rs111950327
rs117630647
rs61405042
rs112394943
rs1023767
rs139669835
rs55747751
rs3767952
rs28502153
rs1907702
rs12451892
rs116863223
rs73730598
rs45604339
rs1957937
rs869785
rs939916
rs6007020
rs10774624
rs77231040
rs188918174
rs4743037
rs9878436
rs16978028
rs17803849
rs113525195
rs376641875
rs6536702
rs6587577
rs450962
rs73581419
rs6584579
rs1003322
rs145114957
rs429358
All
rs2282764
rs4724
rs11212631
rs9940099
rs41304632
rs7221585
rs9398196
rs117407747
rs80116508
rs9600019
rs79977579
rs1611236
rs11769630
rs6790988
rs7164950
rs11085072
rs7209057
rs3785074
rs150150565
rs2977608
rs1332941
rs182059586
rs76065543
rs12369950
rs11579026
rs12932179
rs871134
rs2276182
rs117512405
rs202034370
rs77732866
rs4695407
rs17677991
rs143190905
rs9955360
rs76219171
rs12925933
rs139795227
rs5659669
rs6590343
rs8105767
rs4530278
rs6751209
rs185174247
rs3093888
rs2230590
rs76666449
rs7099229
rs11117354
rs66731853
rs11557154
rs142730696
rs2555104
rs12412214
rs10845387
rs1291143
rs137901416
rs3891167
rs11699829
rs34550383
rs2056726
rs144204502
rs10905255
rs9419958
rs5742915
rs75664430
rs2763979
rs10112752
rs35640778
rs35446936
SNP
-0.025
0.000
0.025
0.050
Effect size (b)
